# Emotional labor and dysmenorrhea in women working in sales and call centers

**DOI:** 10.1186/s40557-014-0045-9

**Published:** 2014-11-07

**Authors:** In-Jung Cho, Hyunjoo Kim, Sinye Lim, Sung-Soo Oh, Sungjin Park, Hee-Tae Kang

**Affiliations:** Department of Occupational and Environmental Medicine, Wonju Severance Christian’s Hospital, Yonsei University, Wonju, South Korea; Department of Occupational and Environmental Medicine, Ewha Womans University Mokdong Hospital, Seoul, South Korea; Department of Occupational and Environmental Medicine, College of Medicine, Kyung Hee University, Seoul, South Korea; Institute of Occupational and Environmental Medicine, Wonju College of Medicine, Yonsei University, Wonju, South Korea

**Keywords:** Emotional labor, Emotive effort, Emotive dissonance, Dysmenorrhea, Sales worker, Call center worker

## Abstract

**Objectives:**

This study was conducted to investigate the association between emotional labor and dysmenorrhea among women working in sales and call centers in Seoul, South Korea.

**Methods:**

Working women in sales jobs and call centers in Seoul were interviewed face-to-face by well-trained interviewers. In total, 975 participants were analyzed in the final model. Emotional labor was measured using a constructed questionnaire with two components: an emotive effort component with three questions and an emotive dissonance component with five questions. To examine the association between emotional labor and dysmenorrhea, chi-squared tests and logistic regression were applied.

**Results:**

The prevalence of dysmenorrhea among sales workers and call center workers were 43.0% and 61.1%, respectively. The adjusted odds ratios (OR) of emotive effort and emotive dissonance for dysmenorrhea in call center workers were 1.88 (95% confidence interval [CI], 1.07–3.28) and 1.72 (95% CI, 1.13–2.63), respectively. The adjusted OR of emotive effort and emotive dissonance for dysmenorrhea in sales workers were 1.71 (95% CI, 0.92–3.16) and 1.15 (95% CI, 0.67–1.98), respectively.

**Conclusions:**

Emotional labor was found to be associated with dysmenorrhea in call center workers. Further studies to investigate other factors, such as management strategies and the relationship between emotional labor and dysmenorrhea, are needed to support interventions to prevent dysmenorrhea that will further promote the quality of health and life of working women.

## Introduction

With the increasing number of working women in South Korea, the population of working women reached 10,518,000, or 42% out all working people, in 2013 [1]. By occupational classification, the types of occupations with a high participation of women are in the service and sales industries. In these two industries, 1,695,000 workers (66%) in service jobs and 1,469,000 (50%) in sales jobs are women [2].

A key difference between these two industries and manufacturing is that manufacturing workers use equipment to produce products, while service and sales workers sell services and goods to customers. In other words, interactions with customers are essential in both the service and sales industries [3]. In *The Managed Heart* (1993), Hochschild defined emotional labor as “the management of feeling to create a publicly observable facial and bodily display” [4]. Jobs requiring emotional labor have three characteristics in common. First, workers come into face-to-face or voice-to-voice contact with customers. Second, workers must show customers emotional responses such as appreciation and fear. Third, employers control employees’ emotional behavior by training and supervision [4]. Hochschild classified emotional management strategies into “surface acting” and “deep acting” [4]. Surface acting involves the management of superficial behaviors, but in deep acting, workers adjust their feelings to fit the demands of the workplace context by relying upon the obligation they feel from their training or the feelings they experienced in previous situations [4]. Building on Hochschild’s theory, Kruml and Geddes suggested a two-dimensional model for emotional labor that divided deep acting into “active deep acting” and “passive deep acting” [5]. The first factor of the two dimensional model, emotive dissonance, is defined as a conflict between feigned and real emotions and exists at a point on the continuum between surface acting and passive deep acting. Emotive effort, which is the next factor, refers to active deep acting to produce the appropriate feelings. Previous studies have reported negative outcomes of emotional labor [5]. According to these studies, emotional labor is associated with physical diseases such as cardiovascular disorders as well as behavioral and emotional problems including exhaustion, job dissatisfaction, low self-esteem, depression, cynicism, and self-alienation [6-11].

Meanwhile, dysmenorrhea is one of the most common gynecological disorders [12-14]. Approximately 50%–90% women have menstrual pain, and an estimated 10%–20% women miss work (absenteeism) or have decreased productivity (presenteeism) [15-17]. Primary dysmenorrhea is defined as severe menstrual pain that can interfere with daily activities despite the absence of other disease involvement during the menstrual period. Various factors including young age [15,17], nulliparity [15,16,18], smoking [15,17-19], a body mass index (BMI) under 20 kg/m^2^, menarche before 12 years of age, a long menstrual period, an irregular and high volume of menses, a sexual assault history [20], mental stress, and job stress [14,21,22] have been identified as risk factors for dysmenorrhea.

Studies on the relationship between emotional labor and dysmenorrhea are rare in Korea and beyond. In this study, the emotional labor level and distribution of dysmenorrhea in working women was investigated. In addition, the association between dysmenorrhea and emotional labor was examined.

## Materials and methods

### Subjects

Well-trained interviewers used a constructed questionnaire from September 10 to October 5, 2012 to convenience sample available, working women in the Geumcheon administrative district of Seoul and surrounding areas. In total, 5,021 interviewees were included (2,459 office workers, 731 call center workers, 603 sales workers, 483 manufacturing workers, 714 workers from other occupations, and 31 workers who did not provide their occupation). Among them, 1,334 worked in sales jobs and/or call centers. Others were excluded because they did not regularly interact with customers. In addition, 126 workers who reported amenorrhea for the past year and 14 workers who did not provide their menstrual schedule for the past year were excluded. After further exclusion for those missing data on emotional labor or other covariates, 975 (73.1%) participants were analyzed. This study did not contain any personal information, therefore ethical approval for this study was waived.

### Survey items

#### Emotional labor

To measure emotional labor, the 8-item scale developed by Chu et al. was applied [23]. This scale classifies the eight items into emotive effort and emotive dissonance using factor analysis [23]. Emotive effort and emotive dissonance are the two components that make up emotional labor. Emotive effort refers to active deep acting to produce the appropriate feelings. Emotive dissonance is defined as a conflict between feigned and real emotions. Three of the eight items represent emotive effort and five represent emotive dissonance [5]. Each item was measured on a four-point scale (1 = never, 4 = strongly agree). To measure emotive effort and emotive dissonance, the sum of points from the three items and five items were used, respectively. The cut-off point for dividing those with high- and low-risk exposure to emotional labor was determined by the distribution of the present study. High-risk exposure groups were operationally defined as those with a score higher than the 75th percentile for all of the subjects. According to this definition, the cut-off point for emotive effort and emotive dissonance was 11 and 15, respectively.

#### Dysmenorrhea

Dysmenorrhea is defined as severe menstrual pain that interferes with daily activities during the menstrual period. In this study, those with a score of 2 points or above were defined as having dysmenorrhea using the four-point scale, which collected data on menstrual pain over the previous three months. One point on this scale represented occasional symptoms or an occasional interruption of daily activities due to menstrual pain. Two points represented considerable pain that was tolerable without medication. Three points represented substantial pain requiring medication to alleviate symptoms. Four points represented pain that persisted even after taking medication.

#### Covariates

Using a constructed questionnaire, risk factors for dysmenorrhea such as age, nulliparity, smoking, BMI, age at menarche, and other general and work characteristics were investigated. The subjects were categorized into three age groups as ≤ 29 years, 30–39 years, and ≥40 years. Subjects’ BMI were also categorized into three groups as < 20 kg/m^2^, 20–25 kg/m^2^, and ≥25 kg/m^2^. In addition, educational background was classified as high school or lower and college and above. In addition, participants were classified as current smokers or non-smokers. A yes or no question was used to determine whether the women lived with her spouse. Instead of measuring parity, residing with children was collected as either yes or no. Moreover, age at menarche was divided into < 12 years or ≥12 years. Average working hours were also categorized into two groups as either < 52 hours per week or ≥52 hours per week.

### Statistical analysis

The chi-squared test was applied to investigate distribution differences in the subjects’ general and occupational characteristics according to the dysmenorrhea and the job type. To examine the distribution differences in the levels of menstrual pain by job classification, a chi-squared test was performed. In addition, chi-squared tests were used to investigate differences between job type and emotive effort or emotive dissonance among each high-risk exposure group, respectively. In addition, median scores and interquartile ranges of the emotive effort and emotive dissonance scores were investigated, respectively. Logistic regression analysis was performed to evaluate the association of emotional labor with dysmenorrhea. Univariate analysis was applied before and after stratification for job type. In addition, emotive effort and emotive dissonance were analyzed after adjustment for age, smoking, educational background, residing with children, and age of menarche. BMI and weekly working hours were not considered covariates that could have affected the risk of dysmenorrhea. In addition, spouse cohabitation was not adjusted for in the regression model since this variable was highly correlated with the variables age and residing with children (data not shown). SAS version 9.2 (SAS Inc., Cary, NC, USA) was used to analyze all data and a p < 0.05 was considered to represent statistical significance.

## Results

Among the 975 total participants, 518 (53.1%) had dysmenorrhea. The prevalences of dysmenorrhea in the age groups ≤ 29 years, 30–39 years, and ≥40 years were 73.8%, 55.1%, and 32.0%, respectively. Thus, younger age was associated with a higher prevalence of dysmenorrhea (*p* < 0.0001). The rate of dysmenorrhea in the smoking group (69.1%) was significantly higher than that in the non-smoking group (48.4%, *p* < 0.0001). When BMI was considered, the percentages of those with dysmenorrhea were 53.5%, 52.7%, and 53.9% among those with a BMI < 20 kg/m^2^, 20–25 kg/m^2^, and ≥25 kg/m^2^, respectively; no significant difference was found among these groups. By educational background, the dysmenorrhea rate among those with a college degree or higher was 58.0%, which was significantly higher than that among those with a high school diploma or lower (50.2%, *p* =0.0203). According to spouse cohabitation, the dysmenorrhea rate among those cohabiting with a spouse was 38.7%, which was significantly lower than that among those not cohabiting with a spouse (65.3%, *p* < 0.0001). The prevalence of dysmenorrhea among those who reached menarche younger than 12 years old was 74.4%, which was significantly higher than that among those who reached menarche at 12 years or older (52.1%, *p* =0.0068). According to the weekly working hours, the dysmenorrhea rate among those working < 52 hours per week was 52.6%, which was not significantly different from the rate among those working ≥52 hours per week (*p* =0.6732) (Table 1).

**Table 1:** **Subjects’ characteristics by dysmenorrhea and job type**

**Variables**		**All**					***p-*** value*	**Sales workers**		**Call center workers**		***p-*** **value** ^**†**^
		**Total**		**Dysmenorrhea**		**Prevalance (%)**		**Total**		**Total**		
		**N = 975**	**(%)**	**n = 518**	**(%)**			**N = 430**	**(%)**	**N = 545**	**(%)**	
Age	≤ 29	309	(31.7)	228	(44.0)	73.8	<.0001^‡^	116	(27.0)	193	(35.4)	<.0001^‡^
	30-39	332	(34.0)	183	(35.3)	55.1		94	(21.9)	238	(43.7)	
	≥ 40	334	(34.3)	107	(20.7)	32.0		220	(51.1)	114	(20.9)	
Smoking	Current smoker	223	(22.9)	154	(29.7)	69.1	<.0001^‡^	81	(18.8)	142	(26.1)	0.0097^‡^
	Non-smoker	752	(77.1)	364	(70.3)	48.4		349	(81.2)	403	(73.9)	
BMI (kg/m2)	< 20	325	(33.3)	174	(33.6)	53.5	0.9599^‡^	175	(40.7)	150	(27.5)	<.0001^‡^
	20 – 24.9	533	(54.7)	281	(54.2)	52.7		243	(56.5)	290	(53.2)	
	≥ 25	117	(12.0)	63	(12.2)	53.8		12	(2.8)	105	(19.3)	
Educational status	High school or lower	608	(62.4)	305	(58.9)	50.2	0.0203^‡^	307	(71.4)	301	(55.2)	<.0001^‡^
	College or higher	367	(37.6)	213	(41.1)	58.0		123	(28.6)	244	(44.8)	
Living with a partner	Yes	445	(45.6)	172	(33.2)	38.7	<.0001^‡^	243	(56.5)	202	(37.1)	<.0001^‡^
	No	530	(54.4)	346	(66.8)	65.3		187	(43.5)	343	(62.9)	
Living with a child	Yes	427	(43.8)	155	(29.9)	36.3	<.0001^‡^	233	(54.2)	194	(35.6)	<.0001^‡^
	No	548	(56.2)	363	(70.1)	66.2		197	(45.8)	351	(64.4)	
Age at menarche (years)	< 12	43	(4.4)	32	(6.2)	74.4	0.0068^‡^	13	(3.0)	30	(5.5)	0.0861^‡^
	≥ 12	932	(95.6)	486	(93.8)	52.1		417	(97.0)	515	(94.5)	
Weekly working hours	≤ 51	643	(65.9)	338	(65.2)	52.6	0.6732^‡^	168	(39.1)	475	(87.2)	<.0001^‡^
	≥ 52	332	(34.1)	180	(34.8)	54.2		262	(60.9)	70	(12.8)	

*Between cases and non-cases.

^†^Between sales workers and call center workers.

^‡^ *p-*value by *χ*2-test.

The prevalence of dysmenorrhea among the high-risk emotive effort group was 67.6%, which was significantly higher than that of the low-risk group (50.7%, *p* =0.0003). In addition, the prevalence of dysmenorrhea among the high-risk emotive dissonance group was 63.5%, which was significantly higher than that of the low-risk group (49.7%, *p* =0.0003). The proportion of high-risk emotive dissonance workers was 24.7%, and this proportion among sales workers was 18.4%, which was significantly lower than that among call center workers (29.7%, *p* < 0.0001). The proportion of high-risk emotive effort workers was 14.6%, and this proportion among sales workers was 13.3%, which was lower than that among call center workers (15.6%); however, there was no statistically significant difference between these two groups (*p* =0.3486) (Table 2).

**Table 2:** **Distribution of dysmenorrhea and emotional distress by job type**

**Variables**			**All**				***p-*** value*	**Sales workers**		**Call center workers**		***p-*** **value** ^**†**^
			**Total**		**Dysmenorrhea**			**Total**		**Total**		
			**N = 975**	**(%)**	**n = 518**	**(%)**		**N = 430**	**(%)**	**N = 545**	**(%)**	
Grade of menstrual pain	1		457	(46.9)	0	0.0		245	(57.0)	212	(38.9)	
	2		258	(26.5)	258	(49.8)		88	(20.5)	170	(31.2)	
	3		204	(20.9)	204	(39.4)		80	(18.6)	124	(22.7)	
	4		56	(5.7)	56	(10.8)		17	(3.9)	39	(7.2)	
Emotive effort	High risk		142	(14.6)	96	(18.5)	0.0003^‡^	57	(13.3)	85	(15.6)	0.3486^‡^
	Median score	(value at 75%, value at 25%)	9	(10,8)	9	(10,8)		9	(10,7)	9	(10,8)	
Emotive dissonance	High risk		241	(24.7)	153	(29.5)	0.0003^‡^	79	(18.4)	162	(29.7)	<.0001^‡^
	Median score	(value at 75%, value at 25%)	12	(14,10)	12	(15,11)		12	(14,10)	13	(15,11)	

*Between cases and non-cases.

^†^Between sales workers and call center workers.

^‡^ *p-*value by *χ*2-test.

Among all workers, results of the crude logistic regression demonstrated that the prevalence of emotive effort and emotive dissonance were both significantly related with dysmenorrhea. The adjusted odds ratio (OR) of emotive effort among all workers with dysmenorrhea was 1.83 (95% confidence interval [CI], 1.22–2.76). The adjusted OR of emotive dissonance among all workers with dysmenorrhea was 1.49 (95% CI, 1.08–2.07).

Among sales workers, results of the crude logistic regression demonstrated that emotive effort is significantly related with dysmenorrhea. The adjusted OR of emotive effort among sales workers with dysmenorrhea was 1.71 (95% CI, 0.92–3.16), which was of borderline statistical significance. Emotive dissonance did not significantly increase the likelihood of having dysmenorrhea among the sales workers.

Among the call center workers, results of the crude logistic regression demonstrated that emotive effort and emotive dissonance are significantly related with having dysmenorrhea. The adjusted OR of emotive effort among call center workers with dysmenorrhea was 1.88 (95% CI, 1.07–3.28). In addition, the adjusted OR of emotive dissonance among call center workers with dysmenorrhea was 1.72 (95% CI, 1.13–2.63) (Table 3).

**Table 3:** **Odds ratios of emotional labor for dysmenorrhea**

**Variables**		**All (N =975)**		**Sales workers (N =430)**		**Call center workers (N =545)**	
		**OR**	**95% CI**	**OR**	**95% CI**	**OR**	**95% CI**
Emotive effort	Crude	2.03	1.39-2.96	2.00	1.14-3.52	2.02	1.20-3.39
	Adjusted	1.83*	1.22-2.76*	1.71^†^	0.92-3.16^†^	1.88^†^	1.07-3.28^†^
Emotive dissonance	Crude	1.76	1.30-2.37	1.29	0.79-2.10	1.85	1.25-2.75
	Adjusted	1.49*	1.08-2.07*	1.15^†^	0.67-1.98^†^	1.72^†^	1.13-2.63^†^

*Model adjusted for age, smoking, education, child, age at menarche, job type.

^†^Model adjusted for age, smoking, education, child, age at menarche.

## Discussion

In this study, 53.1% of our subjects reported suffering from dysmenorrhea. The prevalence of dysmenorrhea among women younger than 30 was 73.8%, which is consistent with the results of previous studies [24,25].

Both factors of emotional labor, emotive effort and emotive dissonance, were significantly associated with dysmenorrhea among all workers. Emotional labor induces stresses [8,26,27] that can cause disorders in the secretions of pulsatile follicle-stimulating hormone and luteinizing hormone and can then impair follicle development [28]. Impaired follicle development induces the synthesis and secretion of progesterone, thus causing an increase in the synthesis of prostaglandin F2-alpha and prostaglandin E2 as well as the binding of prostaglandin to myometrium receptors [29,30]. Therefore, dysmenorrhea has been thought to result from an imbalance of prostaglandin, which controls the tone of the myometrium and the blood vessels [29]. In addition, an increased synthesis of prostaglandin F2 in the myometrial cells due to the stress-related hormone cortisol has been suggested to induce dysmenorrhea [31]. In short, emotional labor might be one of the psychosocial stressors causing dysmenorrhea.

However, the association between emotional labor and dysmenorrhea varied according to occupational type. Both emotive effort and emotive dissonance were significantly associated with having dysmenorrhea among call center workers. Among sales workers, emotive effort was associated with having dysmenorrhea, but with borderline statistical significance. However, there was no statistical significance in the association between emotional dissonance and dysmenorrhea.

A number of factors may explain these results. First, the frequency, period, and intensity of interactions with customers were not investigated in this study. According to a study by Park et al. regarding the frequency of customer interactions, sales workers were found to meet with an average of 32 customers throughout 5.4 hours of work per day, while call center workers were found to speak with an average of 125 customers throughout 5 hours of work per day [32]. Kim et al. found that sales workers meet an average of 100 customers in a typical weekend day, and sales workers were found to spend three to five minutes responding to one customer. In addition, sales workers were found to face double the number of customers during the weekend as compared to the total number of customers they face during the weekdays [33]. However, call center workers receive an average of 550 calls per day [33]. Park et al. reviewed the frequency of emotional events to examine factors related to the intensity of emotional labor in the workplace [32]. They reported that sales and call center workers experienced unreasonable requests from customers 3.6 and 3.9 times, lost face (due to a customer’s rude comments) 1.6 and 3.7 times, and endured violent comments from customers (insulting and cursing) 0.6 and 2.7 times, respectively, during a two-month period [32]. In addition, call center workers experienced sexual harassment an average of 1.1 times over the two-month period [32]. Therefore, call center workers interact with customers more frequently and intensely than sales workers do. Our finding of a stronger association between emotional labor and dysmenorrhea among call center workers supports their findings.

Second, while sales workers communicate with customers face-to-face, call center workers communicate only voice-to-voice; therefore, nonverbal communication is only applicable to sales workers. Nonverbal communication including gestures, eye contact, and touch accounts for two-thirds of typical communication [34]. When there is a lack of nonverbal communication, emotive effort or emotive dissonance can increase. Nonverbal communication in itself can also be a stressor that causes dysmenorrhea. As a result, the lack of nonverbal communication among call center workers can confound and affect the association between emotional dissonance and dysmenorrhea. Third, different job requirements between these two types of jobs could be a reason for the difference in the effect of emotive dissonance on dysmenorrhea between the two groups. Although job decision latitude for call center workers is low [35], sales workers who sell clothing in large-scale outlet stores examined in the present study possessed some job decision latitude. Furthermore, sales workers tend to be people who enjoy working with the public and selling items/services. Thus, the effect of emotive dissonance on dysmenorrhea might be lower among sales workers.

No significant difference in the distribution among the high-risk emotive effort group was found by occupational type; however, the call center workers comprised a higher proportion of workers at a high risk of emotive dissonance. Among the general and occupational characteristics considered in this study, we found no factors related to being at a high risk of emotive dissonance (data not shown). As mentioned above, emotional difficulty, caused by a lack of nonverbal communication, may be an important cause of emotive dissonance.

The limitations of this study are as follows. First, causality between emotional labor and dysmenorrhea cannot be determined due to the cross-sectional design of this study. Second, the participants worked in one area in Seoul; thus, there are limitations to the generalizability of our findings to other sales and call center workers in South Korea. However, we think that the characteristics of the sales workers in our study might be applicable to other sales workers in South Korea because our investigation was conducted on workers from large-scale outlet stores. In addition, because the working processes of call center workers are comparatively similar nationwide, regional variation is not expected to vary widely among different call center workers. Third, we were not able to investigate how many inbound or outbound calls the call center workers received. An inbound call involves responding to a previously submitted inquiry, while an outbound call is assumed to occur without a previous inquiry from the customer. For call center workers, the type of calls, inbound and outbound, may affect their working conditions, types of emotional labor, negative events, and work processes after events [32]. Fourth, severe menstrual pain could be caused by secondary dysmenorrhea due to another primary disease (organopathy or another disease); however, this factor was not excluded from this study.

## Conclusions

Reproductive health is an important issue among sales and service businesses that employ a high proportion of female personnel. Given that research into the effect of emotional labor on reproductive health in sales and service industries are currently rare, it is meaningful to examine the effect of emotional labor on dysmenorrhea. Hochschild’s theory approached the issue from an employee perspective, examining the workers’ emotional labor of deep acting and surface acting. In our study, emotional labor was measured using the model suggested by Kruml and Geddes, which does not take into account the frequency or the period of the interaction with customers. Future studies should be performed with a job-focused approach, in which the frequency, duration, and intensity of interaction with customers and the display rule are investigated [10]. Furthermore, further investigation into potential supporting factors including a stress relief program, professional counseling, and an ombudsman are suggested.

This study found that emotional labor increases the risk of dysmenorrhea in service workers, especially call center workers. Further studies are needed to address the limitations of the present study. Assessment, intervention, and management for the types of jobs showing a high risk for emotional labor and a strong association between emotional labor and dysmenorrhea should be considered.

### Consent

Written informed consent was obtained from the patient for the publication of this report and any accompanying images.
